# Do Children with Autism Spectrum Disorder Share Fairly and Reciprocally?

**DOI:** 10.1007/s10803-018-3528-7

**Published:** 2018-03-06

**Authors:** Calum Hartley, Sophie Fisher

**Affiliations:** 1 Department of Psychology, Lancaster University, Lancaster, LA1 4YF UK; 2 Present Address: Axcis Education, Manchester, UK

**Keywords:** Autism spectrum disorder, Ultimatum Game, Dictator Game, Sharing, Reciprocity, Fairness

## Abstract

This study investigated whether children with autism spectrum disorder (ASD) and typically developing children matched on receptive language share resources fairly and reciprocally. Children completed age-appropriate versions of the Ultimatum and Dictator Games with real stickers and an interactive partner. Both groups offered similar numbers of stickers (preferring equality over self-interest), offered more stickers in the Ultimatum Game, and verbally referenced ‘fairness’ at similar rates. However, children with ASD were significantly more likely to accept unfair offers and were significantly less likely to reciprocate the puppet’s offers. Failure to reciprocate fair sharing may significantly impact on social cohesion and children’s ability to build relationships. These important differences may be linked to broader deficits in social-cognitive development and potentially self-other understanding.

## Introduction

Sharing is a crucial foundation of human evolution (Dunbar 1993; Winterhalder 2001) and involves relinquishing ownership or control of access to a commodity for someone else’s benefit. For decades, behavioural economists have examined the conflict between retaining valued possessions and sharing with others via resource-exchange tasks. In the Ultimatum Game, an individual is endowed with a desirable resource and is required to offer a proportion to a partner who has nothing. On acceptance, the resource is split as proposed and both parties keep a share. On rejection, neither party keeps any of the resource. Thus, the proposer must strategically balance self-interest (i.e. the desire to retain as much of the resource as possible) against their partner’s interests. The Dictator Game follows the same format except for one crucial difference: the partner must always accept whatever share is offered. It is widely argued that players’ responses in these tasks are directed by socially-learned norms concerning fairness (Hoffman et al. 2008) and the ability to infer the mental states of social partners via Theory of Mind (ToM; Castelli et al. 2010; Takagishi et al. 2014). Here, we explore whether children with autism spectrum disorder (ASD)—a population characterised by impairments in social interaction and Theory of Mind (APA 2013; Baron-Cohen 1995)—show differences in resource sharing while playing age-appropriate versions of the Ultimatum and Dictator Games.

According to the economic model of rational self-interest, proposers should always make the smallest possible offers, and responders in the Ultimatum Game should accept any offer greater than zero (Camerer 2003). However, across dozens of studies, typically developing (TD) adults consistently offer 40–45% of the stake in the Ultimatum Game and 20–25% in the Dictator Game (despite having the option to offer less without fear of rejection; Camerer 2003; Henrich et al. 2005; Rigdon 2003). The generosity of these average offers reflects a general preference for fairness and equality. Indeed, adults will usually reject offers they perceive to be unfair, and failure to behave reciprocally elicits punishment and negative affect in exchange partners (Fehr and Gachter 2002; de Quervain et al. 2004). Lucas et al. (2008) investigated whether TD children aged 4–5 years similarly value fairness when sharing endowed commodities. This was achieved by designing age-appropriate versions of the Ultimatum and Dictator Games that employed stickers as a resource (rather than money, tokens, or points), and stakes were distributed immediately after each round (rather than at the end of the task). The results showed that TD children offered 47% and 40% of stakes in the Ultimatum Game and Dictator Game respectively. Therefore, despite the natural desire to retain one’s own resources, even young TD children value fairness over self-interest in sharing contexts (see also Brownell et al. 2013; Castelli et al. 2014).

Many theorists have argued that children’s early-emerging inclination to share equally (and reciprocate others’ sharing behaviours) has adapted to promote cooperation and diminish the impact of self-interests on social cohesion (Hoffman et al. 2008). Upholding shared expectations concerning fairness provides a foundation for positive and reciprocal interactions, and establishes one’s reputation as a good social partner (which may be a stronger motivating factor in typical development than greater material or instrumental outcomes; Adamson et al. 2010; Dawson et al. 2004; Greene et al. 2011; Hoffman et al. 2008). From 3-years, TD children display strong adverse reactions when they are disadvantaged by unequal distributions (despite showing little willingness to share themselves; LoBue et al. 2011). By 4-years, TD children can infer the emotions, needs, and interests of social partners, and are able to differentiate these from their own (Wellman et al. 2001). At 5-years, they make explicit verbal references to fairness, demonstrate a motivation to engage in behaviour that benefits others, and show generosity when sharing resources with partners (Fehr et al. 2008; Güroğlu et al. 2009; Lucas et al. 2008). Thus, TD children may offer nearly half of a valued resource in the Ultimatum Game because they can represent the perspective of the responder and are aware that a lower offer may be construed as “unfair”. In support of this reasoning, TD children with superior ToM skills make higher mean offers and are more likely to reject unfair offers in the Ultimatum Game (Castelli et al. 2010; Takagishi et al. 2010, 2014). Taken together, this evidence suggests that TD children’s preference for sharing fairly in resource-exchange tasks is driven by sensitivity to social norms and awareness of others’ perspectives.

If the development of equal sharing is underpinned by social norms and awareness of others’ mental states, we may expect to observe qualitative differences in ASD. Children with ASD show diminished social motivation and experience difficulties interacting with others (APA 2013; Chevallier et al. 2012). Compared with TD children, those with ASD spend less time engaged in social interactions with peers (Bauminger et al. 2008), are less likely to collaborate (Aldridge et al. 2000; Carpenter et al. 2001; van Ommeren et al. 2012), and are less likely to reciprocate in naturalistic interactions (Channon et al. 2001; Hadwin et al. 1997; Wimpory et al. 2007; Joseph and Tager-Flusberg 2004; Klin et al. 2006; Ozonoff and Miller 1995). It is also widely acknowledged that children with ASD have fundamental impairments in intention reading and ToM (Baron-Cohen 1995; Baron-Cohen et al. 1997; Charman et al. 1997; D’Entremont and Yazbek 2007; Griffin 2002; Hartley and Allen 2014, 2015; Hobson 2002; Mundy and Willoughby 1996; Preissler and Carey 2005). These deficits result in reduced understanding and consideration of others’ psychological states both separately and in relation to one’s own interests. Theoretically, it is possible that these social-cognitive difficulties impact children’s preferences for fairness and reciprocity when sharing resources. Indeed, it may be that sharing in children with ASD is primarily motivated by instrumental outcomes, and is influenced less by the behaviours and mental states of social partners (Schmitz et al. 2015).

To date, few studies have investigated the sharing behaviour of children with ASD using resource-exchange tasks. In Sally and Hill (2006), high-functioning children with ASD aged 6–15 years played computerised versions of the Ultimatum and Dictator Games, in which ‘points’ served as proxies for real resources. While children with ASD made similar offers to TD controls in the Dictator Game, the groups diverged in the more strategic Ultimatum Game. Whereas most TD children shared the resource equally, many children with ASD—particularly those who failed a false belief test—tended to offer one or zero points (out of ten). Furthermore, when offered 30% or less of the total stake, children with ASD accepted on approximately 30% of trials, whereas TD controls accepted on just 11%. In another study, Schmitz et al. (2015) tested “cognitively able” children with ASD and TD controls aged 9–14 years on a computerised version of the Dictator Game in which they decided how to distribute coins between themselves and an anonymous partner. Crucially, children could choose either an equal distribution (1 point each) or an unequal distribution that benefited either the participant (2 vs 1) or the partner (1 vs 2). Although both populations tended to select the equal split, children with ASD were more likely to select unequal distributions of either type. Recently, in Paulus and Rosal-Grifoll (2016), 3–6 year old children with ASD and TD controls matched on non-verbal ability were tasked with sharing resources with partners that were rich or poor. Unlike TD children who consistently split the resources equally between parties, children with ASD allocated most of the resources to the other recipients and kept relatively little for themselves. The findings from these three studies suggest that children with ASD have a diminished aversion to inequity and are less concerned about their own gains. Furthermore, their sharing tends to maximize resources across parties, accommodating both advantageous and disadvantageous inequality.

Atypical sharing behaviour and weaker preferences for equality could have important implications for children’s social relationships. Specifically, these characteristics may place children with ASD at increased risk of bullying. Recent estimates suggest that up to 87% of children with ASD are bullied every week or month, placing them at significantly higher risk than TD children (Cappadocia et al. 2012; Wainscot et al. 2008). Due to their socially incongruent behaviour and difficulties conforming to social norms, children with ASD are often perceived as ‘different’ by their peers (Humphrey and Lewis 2008; van Roekel et al. 2010). This can impact their ability to develop friendships (Bauminger and Kasari 2000; Chamberlain et al. 2007), leading to feelings of isolation and increasing the likelihood of victimisation (Bauminger et al. 2003; Hodges et al. 1999; Humphrey and Symes 2011). If children with ASD are more receptive to unfair social behaviour and less concerned about their personal gain, this could significantly increase their risk of exploitation or manipulation.

The objective of this study was to explore the sharing behaviour of children with ASD and language-matched TD controls via age-appropriate versions of the Ultimatum Game and Dictator Game. In doing so, we advance the literature in three important ways. Firstly, prior studies have relied upon computer-based tasks that involve sharing “virtual resources” with hypothetical or inanimate partners. Lucas et al. (2008) point out that children may not understand that points represent commodities, and may behave differently when required to share tangible rewards with real partners. Thus, we increased the stakes of sharing by endowing children with attractive stickers (a valued resource often used to reward and reinforce positive behaviour in both populations), and instructing them to share with a pseudo-animate partner (a puppet) in a face-to-face context. Secondly, we explored how children’s offers are influenced by the offers of their partner. Previous studies document children’s offers and responses, but do not test the extent to which children with ASD *reciprocate* fair or unfair offers. Exploring this behaviour will provide an indication of children’s sensitivity to the fairness norm and their ability to adapt to others’ behaviour. Thirdly, the rationale underpinning the sharing behaviours of children with ASD is currently unknown. We shed light on this motivation by recording and analysing children’s verbal justifications of their offers and responses when resources are distributed. In addition, we conducted an ‘unexpected contents’ false belief task to establish whether ToM relates to sharing behaviour. Based on previous resource exchange studies (Sally and Hill 2006; Schmitz et al. 2015) and evidence of reduced social reciprocity (e.g. Klin et al. 2006), we expected to observe a diminished preference for equality, reduced reciprocation of fair offers, and fewer verbal references to “fairness” in children with ASD. In comparison to previous studies, we anticipated that the increasingly social context and real-life rewards may heighten self-interest in the ASD group.

## Method

### Participants

Participants were 15 verbal children with ASD (13 male; *M* age = 9.2 years, range 7.1–11.1 years) and 18 TD children (12 male; *M* age = 4.3 years, range 3–6.1 years) recruited from two specialist schools and one mainstream school in Cheshire, UK. As cognitive development in ASD is often delayed relative to chronological age (Anderson et al. 2009), we adopted Sally and Hill’s (2006) approach of matching samples on language comprehension rather than chronological age (allowing us to assume with reasonable confidence that participants in both groups could understand the task). Samples were closely matched on receptive vocabulary as measured by the British Picture Vocabulary Scale (BPVS; ASD: *M* age equivalent: 5.1 years, *SD*: 1.67; TD: *M* age equivalent: 4.83 years, *SD*: 1.59; Dunn et al. 1997). As every child with ASD had delayed linguistic development in comparison to their chronological age, our sample is representative of a significant proportion of the clinical population (Anderson et al. 2007). All children with ASD were diagnosed by a qualified educational or clinical psychologist, using standardised instruments (i.e. Autism Diagnostic Observation Scale and Autism Diagnostic Interview—Revised; Lord et al. 2002, 1994) and expert judgement. Diagnoses were confirmed via the Childhood Autism Rating Scale (CARS; Schopler et al. 1980), which was completed by each participant’s class teacher (ASD: *M* score = 31.78; TD: *M* score = 15.42). Children with ASD were significantly older (*t*(31) = 13.24, *p* < .001, *d* = 4.52), and had significantly higher CARS scores (*t*(31) = 8.48, *p* < .001, *d* = 3.7) than the TD children. The study was approved by the Lancaster University Ethics Committee, and informed consent was obtained from children’s caregivers prior to their involvement in the research.

### Materials

Following Lucas et al. (2008), brightly-coloured stickers were used as trading items in the Ultimatum and Dictator Games as they are desirable and often used as positive reinforcers. Every child was presented with eight sets of eight stickers (one set per trial of each game). The sticker sets were different from one another in order to maintain interest and motivation throughout each game (e.g. smiley faces, animals, stars etc). However, within a set, stickers were thematically similar (e.g. differently coloured stars) to reduce the likelihood that children would develop strong preferences for individual stickers that would impact their willingness to trade. In line with previous studies of this nature, children interacted with a human-looking hand puppet that matched their gender (“Jack” or “Jill”) during the experimental tasks (e.g. Kanngiesser and Hood 2014). Children were unlikely to view the puppet as an authority figure, meaning their trading decisions would not be influenced by unequal status.

For the Unexpected Contents Task, a Smarties tube was emptied and filled with small colouring pencils. Three pictures were created to facilitate the responding of children with ASD if necessary (depicting a tube of Smarties, colouring pencils, and a rainbow).

### Procedure

Participants were tested individually in their own schools and were accompanied by a familiar adult. Children were verbally praised for attention and good behaviour. Participants completed three test sessions on different days. Session 1 consisted of the BPVS. Session 2 involved the Ultimatum or Dictator Game (counterbalanced across participants). Session 3 involved either the Ultimatum or Dictator Game (whichever was not played in Session 2) followed by the Unexpected Contents Task.

#### Ultimatum Game

The Ultimatum Game consists of two roles: proposer and responder. The roles alternated between the child and puppet over four trials (e.g. the child was the proposer for trials 1 and 3). Half of the participants started in the proposer role, while the other half started in the responder role. When in the proposer role, the child was given eight stickers (per trial) and instructed to give some to the puppet, with a one sticker minimum offer. If the puppet accepted the offer, the stickers were divided as proposed. If the puppet rejected, neither player received any stickers. When in the responder role, the child accepted or rejected an offer from the puppet. Acceptance lead to both parties receiving stickers while rejection meant neither party received any stickers. The puppet offered one sticker (unfair offer) on one trial and four stickers (fair offer) on another trial (order randomly predetermined). The puppet accepted one of the child’s offers and rejected the other (order randomly predetermined). After making their offers, children were asked why they had made this decision (“Why did you give Jack that number of stickers?”). They were also asked how they felt about each of the puppet’s offers (“Do you want that many stickers? Why?”).

#### Dictator Game

This game followed the same procedure as the Ultimatum Game, except the responder was unable to reject the proposer’s offer. The proposer role alternated between the child and puppet over four trials (e.g. the child was the proposer for trials 1 and 3). Half of the participants started in the proposer role, while the other half received the puppet’s offer first. As the proposer, children were given eight stickers (on each trial) and instructed to give some to the puppet, with a one sticker minimum offer. They were informed that the puppet had to accept their offer (e.g. “Jack has to take the number of stickers you give him”). The puppet offered 1 sticker (unfair offer) on one trial and four stickers (fair offer) on another trial (order randomly predetermined). Children were asked to explain their offers, and describe how they felt about the puppet’s offers.

#### Unexpected Contents Task

The puppet was hidden from view at the start of this task (they were “sleepy and needed a nap”). Children were shown a Smarties tube and asked what they thought was inside. The tube was opened to reveal small coloured pencils instead of Smarties. The pencils were placed back inside the Smarties tube and the puppet “woke up”. Children were then asked three questions in a random order: (a) “what does Jack/Jill think is inside?”, (b) “what did you think was inside when you first saw it?”, and (c) “what is really inside?” (a memory check to identify children who were guessing or did not understand). Children with ASD who had limited expressive language responded to each question by pointing to one of three colour pictures depicting a tube of Smarties, colour pencils, and a rainbow (to control for guessing).

## Results

When children were in the proposer role, we recorded the number of stickers they offered the puppet on each trial. In the Ultimatum Game we recorded whether children accepted or rejected each of the puppet’s offers and we also recorded children’s verbal comments in both games.

### Ultimatum Game

#### Children’s Offers

On average, children with ASD offered 2.93 (*SD*: 1.22; 36.63% of the total stake) stickers on their first turn in the proposer role, and 3.53 (*SD*: 1.77; 44.13%) stickers on their second turn. By comparison, TD children offered 3.72 (*SD*: 2.22; 46.5%) stickers on their first turn as proposer, and 3.06 (*SD*: 1.39; 38.25%) on the second. These data were entered into a two (population: TD, ASD) × 2 (offer: first, second) mixed ANOVA, which revealed no significant effects. Thus, an ASD diagnosis did not significantly impact first or second offers made by children in the Ultimatum Game.

We then tested whether children’s offers were influenced by their starting role: proposer or responder. Data from each population were entered into a 2 (order: child first, puppet first) × 2 (offer: first offer, second offer) mixed ANOVA. These analyses revealed no effects, suggesting that neither group’s offers were influenced by whether the participant started in the proposer or responder role. To assess whether the populations differed when making initial offers without a prior cue (e.g. a preceding offer from the puppet) we conducted an independent samples *t* test. The results confirmed that the first offers of TD children and children with ASD who started in the proposer role did not significantly differ in the Ultimatum Game.

While the analyses of children’s numerical offers have not revealed any significant differences between populations, it is important to note that they do not consider the influence of the puppet’s behaviour. Reciprocity is a vital aspect of sharing and we were interested to discover whether the fairness of children’s offers was influenced by the fairness of the puppet’s offers. When the puppet made a fair offer, TD children responded with a fair offer 93% of the time, or an offer that favoured themselves 7% of the time. When the puppet made an unfair offer, TD children responded with a fair offer 36% of the time, an offer that favoured themselves 64% of the time, and never made offers that favoured the puppet. Thus, the offers of TD children appear to be strongly mediated by the puppet’s behaviour; when they received a fair or unfair offer, they responded in kind on nearly 80% of trials. When children with ASD received a fair offer from the puppet, they responded with a fair offer 56% of the time, or an offer that favoured themselves 44% of the time. When the puppet made an unfair offer, children with ASD responded with a fair offer 25% of the time, an offer that favoured themselves 42% of the time, and an offer that favoured the puppet 33% of the time. These frequencies suggest that the children with ASD were less likely to reciprocate the puppet’s actions compared to TD children; they reciprocated fair and unfair offers just 49% of the time.

We tested whether children with ASD were statistically less likely to reciprocate the puppet’s offers in the Ultimatum Game via a generalized linear mixed-effects model (GLMM). The analysis modelled the probability (log odds) of children reciprocating the puppet’s offer (yes/no), considering variation across participants (random intercepts), fixed effects of population (ASD/TD) and puppet’s offer (fair/unfair), plus the interaction between these variables. We conducted a sequence of GLMMs, entering fixed effects simultaneously. Model 1 was a “null model” containing only the random effect of participant ID. Model 2 added main effects of population and puppet’s offer. Model 3 then added the population × puppet’s offer interaction. We evaluated the relative utility of each increasingly-complex model using likelihood ratio tests. These indicated that inclusion of the main effects in Model 2 yielded a significant improvement in fit over the null model, χ^2^ (2) = 8.16, *p* = .017. Adding the interaction afforded no further improvement. Therefore, Model 2 provides the best fitting explanation of the observed data (see Table 1). In support of our hypotheses, the results show that children with ASD were significantly less likely than TD controls to reciprocate the puppet’s offers in the Ultimatum Game (49% vs 78.5%). However, across populations, there was no difference in reciprocation rates for fair or unfair offers made by the puppet.

**Table 1:** Summary of the final generalized linear mixed-effects model (log odds) of children’s offer reciprocation in the Ultimatum Game as predicted by population (ASD, TD) and puppet’s offer (fair, unfair); note that the ASD group and puppet’s fair offer are taken as reference levels

Fixed effects	Estimated coefficient	Std. error	Z	Pr(>|z|)
(Intercept)	0.55	0.6	0.91	0.36
Population (TD)	1.43	0.69	2.09	0.04
Puppet’s offer (unfair)	− 1.13	0.69	− 1.64	0.1

	AIC	BIC	logLik	Deviance
	59.3	66.6	− 25.6	51.3

*AIC* Akaike information criterion; *BIC* Bayesian information criterion; *logLik* log-likelihood; Pr(>|z|), probability/statistical significance

#### Children’s Responses

Next, we explored how children *responded* to the puppet’s fair and unfair offers. For each population, the relationship between the puppet’s offers (fair offer, unfair offer) and children’s responding (accept, reject) was measured via a McNemar test. The responses of TD children were significantly mediated by the fairness of the puppet’s offer, *p* < .001. They accepted 94% of fair offers and 11% of unfair offers made by the puppet. The responses of children with ASD were also mediated by the fairness of the puppet’s offer, *p* = .016. They accepted 100% of fair offers and 40% of unfair offers. These data suggest that both groups were overwhelmingly biased towards accepting the puppet’s offer of four stickers (likely recognising it as fair), but the children with ASD were nearly 30% more likely than the TD children to accept the puppet’s unfair offer of one sticker. The significance of this difference was tested by examining the relationship between population (TD, ASD) and children’s responding (accept, reject) to fair and unfair offers *separately*. For unfair offers, a chi square test of independence revealed a borderline relationship, χ^2^ (1, *N* = 33) = 3.72, *p* = .054, φ = .34, suggesting that children’s responding was mediated by their diagnostic group. By contrast, there was no relationship between population and children’s responding to fair offers. These results suggest that the two populations have similar sensitivity and response patterns when a partner shares fairly, but their reactions differ when a partner shares unfairly.

### Dictator Game

On average, children with ASD offered 2.87 (*SD*: 1.55; 35.88% of the total stake) stickers on their first turn in the proposer role, and 2.67 (*SD*: 1.4; 33.38%) stickers on their second turn. By contrast, TD children offered 2.44 (*SD*: 1.25; 30.5%) stickers on their first turn as proposer, and 3.06 (*SD*: 1.31; 38.25%) on the second. As for the Ultimatum Game, a 2 (population: TD, ASD) × 2 (offer: first, second) mixed ANOVA revealed no main effects or interaction, indicating no significant differences between the first and second offers of either group. Similarly, a pair of 2 (order: child first, puppet first) × 2 (offer: first offer, second offer) mixed ANOVAs demonstrated that neither group was influenced by starting role when making offers in the Dictator Game. We then examined whether the populations differed when making initial offers without a prior cue (e.g. a preceding offer from the puppet). The results of the independent samples *t* test indicated that the first offers of TD children and children with ASD who started in the proposer role did not significantly differ in the Dictator Game.

As above, we examined the reciprocity of children’s offers. When the puppet made a fair offer, TD children responded with a fair offer 75% of the time, an offer that favoured themselves 19% of the time, or an offer that favoured the puppet 6% of the time. When the puppet made an unfair offer, TD children responded with an offer that favoured themselves 100% of the time. As in the Ultimatum Game, the offers of TD children were apparently influenced by the puppet’s behaviour; they reciprocated fair and unfair offers on 84% of trials. Opposite to the Ultimatum Game, TD children were 25% more likely to reciprocate unfair offers than fair offers. For children with ASD, when the puppet made a fair offer, they responded with a fair offer 50% of the time, an offer that favoured themselves 40% of the time, or an offer that favoured the puppet 10% of the time. When the puppet made an unfair offer, children with ASD responded with a fair offer 10% of the time, an offer that favoured themselves 80% of the time, and an offer that favoured the puppet 10% of the time. Thus, children with ASD reciprocated the puppet’s offers on 65% of trials overall.

A GLMM was constructed to test whether children with ASD were statistically less likely to reciprocate the puppet’s offers in the Dictator Game. The analysis modelled the probability (log odds) of children reciprocating the puppet’s offer (yes/no), considering variation across participants (random intercepts), fixed effects of population (ASD/TD) and puppet’s offer (fair/unfair), plus the interaction between these variables. We conducted a sequence of GLMMs, entering fixed effects simultaneously. Model 1 was a “null model” containing only the random effect of participant ID. Model 2 added main effects of population and puppet’s offer. Model 3 then added the population × puppet’s offer interaction. Likelihood ratio tests were conducted to assess the relative utility of each model. These showed that inclusion of the main effects in Model 2 yielded a significant improvement in fit over the null model, χ^2^ (2) = 8.47, *p* = .015. Adding the interaction afforded no further improvement. Therefore, Model 2 provides the best fitting explanation of the observed data (see Table 2).

**Table 2:** Summary of the final generalized linear mixed-effects model (log odds) of children’s offer reciprocation in the Dictator Game as predicted by population (ASD, TD) and puppet’s offer (fair, unfair); note that the ASD group and puppet’s fair offer are taken as reference levels

Fixed effects	Estimated coefficient	Std. error	Z	Pr(>|z|)
(Intercept)	− 0.16	0.6	− 0.27	0.79
Population (TD)	1.4	0.79	1.78	0.07
Puppet’s offer (unfair)	2.03	0.9	2.26	0.02

	AIC	BIC	logLik	Deviance
	51.7	59.3	− 21.9	43.7

*AIC* Akaike information criterion; *BIC* Bayesian information criterion; *logLik* log-likelihood; Pr(>|z|), probability/statistical significance

The results revealed a borderline effect of population, suggesting that children with ASD tended to reciprocate the puppet’s fair and unfair offers less frequently. There was also a highly-significant effect of puppet’s offer; across populations, children were significantly more likely to reciprocate unfair offers (90%) than fair offers (62.5%). Viewed alongside the opposing trend in the Ultimatum Game (74% fair vs 53% unfair), these results suggest that children moderated their reciprocity strategically overall. That is, they were more likely to reciprocate fair or unfair sharing depending on whether selfish behaviour could, or could not, be penalised by the responder. However, in contrast to this general trend, there was very little difference between reciprocation rates for fair offers by children with ASD in the Dictator Game and Ultimatum Game (50% vs 56%).

### Ultimatum Game Versus Dictator Game

We assessed children’s strategic resource allocation by making direct comparisons between offers on the Ultimatum and Dictator Games. We began by testing the interaction between diagnosis and game type by entering children’s offers into a 2 (population: TD, ASD) × 2 (game: ultimatum, dictator) × 2 (offer: first, second) mixed ANOVA. There was a significant main effect of game, *F*(1, 31) = 8.58, MSE = 1.17, *p* = .006, η_p_^2^ = .22, indicating that both TD children and children with ASD made larger average offers in the Ultimatum Game (ASD *M*: 3.31; TD *M*: 3.39) than in the Dictator Game (ASD *M*: 2.77; TD *M*: 2.75). These results show that both populations adjusted the size of their offers in accord with the different game rules. There was also a significant population × game × offer interaction, *F*(1, 31) = 6.37, MSE = 1.39, *p* = .017, η_p_^2^ = .17. To establish the cause of the three-way interaction, separate 2 (game) × 2 (offer) repeated measures ANOVAs were conducted on the data for each population. For children with ASD, there was a significant main effect of game, *F*(1, 14) = 5.91, MSE = 0.55, *p* = .029, η_p_^2^ = .3, confirming that offers in the Ultimatum Game were greater than offers in the Dictator Game. There was no effect of offer and no interaction. For TD children, a significant main effect of game was qualified by a significant game × offer interaction, *F*(1, 17) = 4.4, MSE = 1.67, *p* = .05, η_p_^2^ = .21, which was explored via a series of Bonferroni-adjusted pairwise tests. First offers in the Ultimatum Game (*M*: 3.72) were significantly larger than first offers in the Dictator Game (*M*: 2.44), *t*(17) = 2.36, *p* = .03, *d* = .59. The difference between second offers was not significant, nor were the differences between first and second offers within either the Ultimatum Game or Dictator Game.

### Verbal Responses

The verbal responses provided by participants during the Ultimatum and Dictator Games were transcribed and a coding scheme was developed. Children’s comments were first categorised based on *context* [(1) following their offer, (2) in response to a fair offer from the puppet, (3) in response to an unfair offer from the puppet] and then allocated to a sub-category based on their *content* (see Table 3). The purpose of this coding system was to identify whether children with ASD and TD children differ in *how* they justify their behaviour in different situations (e.g. by explicitly referring to fairness at different frequencies). Every comment was coded by the second experimenter and an independent rater with relevant expertise. The second rater was blind to the objectives of the study and the details of each child (e.g. their age, population, background scores). Reliability of the coding categories for each context was assessed via Cohen’s Kappa, which was calculated based on the two raters’ categorical classifications. High inter-rater reliability was achieved for all contexts (following child’s offer: κ = .88, *p* < .01; response to fair offer: κ = 1.00, *p* < .01; response to unfair offer: κ = .86, *p* < .01). Disagreements in classifications were resolved by consensus between the two raters.

**Table 3:** Coding scheme for children’s qualitative comments in the Ultimatum and Dictator Games

Context	Category	Definition	Example
Following child’s offer	Simple	Justifies offer without reference to ownership or fairness/sharing	“Just a good amount”
	Fairness	Justifies offer with an explicit reference to a notion of fairness/sharing	“He gave half, I give half”
	Ownership/selfishness	Justifies offer with reference to ownership or wanting more than the puppet	“I want to keep all my ones”
	Prosocial offer	Indicates they were trying to elicit a positive emotional state in their partner	“Make him happy with that many”
Response to fair offer	Positive simple	Expresses satisfaction with reference to emotion or number	“That’s a good number”
	Negative simple	Expresses dissatisfaction with reference to emotions or number	“Not happy”
	Fairness	Explicit reference to a notion of fairness/sharing	“Happy with even amount”
Response to unfair offer	Positive simple	Expresses satisfaction with reference to emotion or number	“I want this one”
	Negative simple	Expresses dissatisfaction with reference to emotions or number	“I wanted more”
	Fairness	Explicit reference to a notion of fairness/sharing	“I want same amount, is really not fair”

Frequencies of response types made by TD children and children with ASD are shown in Table 4. Chi square tests of independence showed that response types in each context were not mediated by population [following child’s offer: χ^2^ (3, *N* = 64) = 2.30, *p* = .51; response to fair offer: χ^2^ (2, *N* = 66) = .90, *p* = .64; response to unfair offer: χ^2^ (2, *N* = 66) = 3.58, *p* = .17].

**Table 4:** Frequencies of qualitative response types made by TD children and children with ASD in the Ultimatum and Dictator Games

Context	Category	Population	
		ASD	TD
Following child’s offer	Simple	2 (7%)	3 (9%)
	Fairness	13 (43%)	19 (56%)
	Ownership/selfishness	6 (20%)	7 (21%)
	Prosocial offer	9 (30%)	5 (14%)
Response to fair offer	Positive simple	18 (60%)	20 (55%)
	Positive negative	0 (0%)	1 (3%)
	Fairness	12 (40%)	15 (42%)
Response to unfair offer	Positive simple	7 (23%)	3 (8%)
	Positive negative	15 (50%)	25 (69%)
	Fairness	8 (27%)	8 (23%)

### Unexpected Contents

All children correctly answered the memory check correctly (“what is really inside [the Smarties tub]?”). Children scored 0–2 based on how many Theory of Mind questions they answered correctly. Mean scores for the children with ASD and TD children were 0.59 and 1.33 respectively, a significant difference, *t*(31) = 2.34, *p* = .026, *d* = .82. It is noteworthy that 65% of the ASD group answered both Theory of Mind questions *incorrectly* (compared with 28% of the TD group), indicating their difficulty understanding their own and others’ mental states.

The influence of children’s Theory of Mind task performance on their offers in the Ultimatum/Dictator Games was examined. Children were assigned to a ‘fail’ category if they answered both unexpected contents test questions incorrectly or a ‘pass’ category if they answered at least one test question correctly (further sub-dividing participants based on one or two correct answers would have resulted in insufficient sample sizes). Children’s offers in the Ultimatum Game and Dictator Game were entered into a pair of 2 (population: TD, ASD) × 2 (Theory of Mind: pass, fail) × 2 (offer: first, second) mixed ANOVAs. The analysis for the Ultimatum Game revealed a significant Theory of Mind × offer interaction, *F*(1, 29) = 4.78, MSE = 2.42, *p* = .037, η_p_^2^ = .14, which was explored using Bonferroni-adjusted pairwise tests. Children who failed both Theory of Mind test questions made significantly smaller first offers (*M* = 2.57) than those who passed at least one (*M* = 3.95), *t*(31) = 2.24, *p* = .032, *d* = .82. However, the second offers made by the pass and fail groups did not differ. The ‘fail’ group showed an almost-significant tendency to make larger second offers (*M* = 3.64) than first offers (*M* = 2.57), *t*(13) = 2.03, *p* = .06, *d* = .55, while the ‘pass’ group showed a non-significant trend in the opposite direction (first offer *M* = 3.95; second offer *M* = 3.00; *t*(18) = 1.8, *p* = .09, *d* = .42). No other main effects or interactions were significant.

The analysis for the Dictator Game revealed no main effects or interactions. Taken together, these findings indicate that Theory of Mind (rather than ASD) influences children’s opening offers in the Ultimatum Game, but not the relatively less strategic Dictator Game.

## Discussion

This study compared how children with ASD and language-matched TD controls shared resources in age-appropriate versions of the Ultimatum Game and Dictator Game. In contrast to previous ASD research, children were required to share real stickers—a tangible and desirable commodity—with an interactive partner in a face-to-face context. In addition to measuring their offers and responses, we also examined children’s tendency to reciprocate the puppet’s behaviour, and recorded their qualitative comments in a variety of situations. The results revealed many similarities in the way that TD children and children with ASD played the resource exchange games; both groups indicated a preference for equality over self-interest when making offers, they offered more stickers in the Ultimatum Game than the Dictator Game, and they explicitly referred to ‘fairness’ at similar rates. However, we observed important between-group differences in reciprocity that suggest ASD impacts children’s ability to modify their sharing based on others’ behaviour.

When required to share stickers with a partner, Lucas et al. (2008) found that TD children aged 4–5 years demonstrated a preference for equality by offering 47% of their stake in the Ultimatum Game and 40% in the Dictator Game. In the present study, TD children aged 3–6 years offered 42% of their stake in the Ultimatum Game and 34% in the Dictator Game. Surprisingly, children with ASD made very similar average offers of 40% and 35% in the Ultimatum and Dictator Games respectively. The two groups also explicitly commented about fairness at similar rates when making and responding to offers. These results support those of Sally and Hill (2006) and oppose the theory that sharing in ASD is increasingly governed by self-interest. Thus, despite the natural desire to retain one’s own material possessions, the offers of TD children and children with ASD do not align with the economic model of rational self-interest (Camerer 2003).

Many studies have posited that fair and reciprocal sharing is underpinned by the ability to represent and understand others’ intentions, emotions, and perspectives (Brownell et al. 2013; Castelli et al. 2014; Lucas et al. 2008; Schelling 1960). Although many children in the ASD group showed impaired ToM (65% failed both questions in the false belief task), this deficit did not influence the average value of their offers. Our results showed that children across both populations who failed both false belief questions tended to make significantly smaller first offers in the Ultimatum Game than peers who answered at least one question correctly. This may suggest that children who are yet to develop ToM are less concerned about making a positive impression at the start of the interaction that would establish their reputation as a good social partner. By contrast, children with more sophisticated understanding of mental states may be increasingly mindful that acting in their partner’s interests is likely to promote a cooperative and cohesive interaction.

Although the average offer values did not differ between populations, we observed several important indicators that ASD affects children’s ability to evaluate the fairness of others’ sharing behaviours and to reciprocate accordingly. While both groups were heavily biased towards accepting the puppet’s fair offers in the Ultimatum Game, children with ASD were almost 30% more likely than TD children to accept unfair offers. This finding replicates Sally and Hill (2006), and aligns with previous observations that children with ASD prefer resource allocations that maximise benefits across parties (Schmitz et al. 2015). One explanation for this behaviour is that deficits in social-cognition (e.g. Chevallier et al. 2012) cause children with ASD to be less concerned about defending norms associated with reciprocal and cooperative interaction. Consequently, these children might be increasingly motivated by instrumental outcomes, irrespective of whether they are personally advantaged or disadvantaged (Paulus and Rosal-Grifoll 2016; Schmitz et al. 2015). To a child with ASD, accepting an unfair offer may be favourable because it yields a greater physical reward than rejection. Thus, the responses of children with ASD indicate an approach to sharing that is characterized by reduced interest in social-relational outcomes and diminished aversion to inequity. By contrast, TD individuals almost always reject unfair offers because of their strong preference for equality and their desire to establish a mutually-beneficial and cooperative relationship (Fehr and Gachter 2002; Hoffman et al. 2008; Lucas et al. 2008).

Intriguingly, in the Ultimatum Game, children with ASD were 37% less likely to reciprocate fair offers and 22% less likely to reciprocate unfair offers. This significant between-population difference clearly indicates that children with ASD did not adapt their behaviour in accordance with the puppet’s. Children with ASD also showed reduced reciprocation in the Dictator Game, and both groups were significantly more likely to reciprocate unfair offers than fair offers in this context. It would appear that both groups realised that the power imbalance enabled them to reciprocate self-interest oriented behaviour without fear of consequence. By contrast, both groups were more hesitant to reciprocate unfair offers in the Ultimatum Game, presumably recognising that the partner still needed to be appeased (despite their selfish behaviour) in order to gain stickers. In this more socially-strategic context, TD children reciprocated 93% of the puppet’s fair offers, clearly indicating their adherence to the cultural norm of fairness and their concern for keeping the puppet “onside”. By contrast, children with ASD demonstrated much lower, and highly similar, reciprocation rates for fair offers in both the Ultimatum Game (56%) and Dictator Game (50%). This striking finding highlights an interesting conundrum: children with ASD may possess and exercise an explicit notion of fairness (as indicated by their offer values and comments), yet it does not appear to be informed by others’ prosocial behaviour.

While children with ASD may learn a ‘fairness heuristic’ that generally privileges equality (Sally and Hill 2006), we propose that fundamental deficits in social-cognition and interaction may diminish the perceived importance of reciprocal fairness. This is epitomised by their failure to recognise the strategic importance of reciprocal fair sharing in the Ultimatum Game. It is theorised that TD children’s inclination to reciprocate fair behaviour serves to promote cooperation, social cohesion, and foster mutually beneficial relationships (Hoffman et al. 2008). These positive interpersonal outcomes may be less important to children with ASD due to their reduced social motivation and impaired ability to represent others’ mental states (Baron-Cohen 1995; Chevallier et al. 2012). Alternatively, differences in reciprocity when sharing may be related to impaired self-understanding in ASD (Frith 2003; Lind 2010). Typically, as a child’s understanding of the self develops, so too does their understanding of others (Moore 2007). Children with greater self-understanding may be better able to reflect and act on the needs of others by drawing comparisons with their own situation and experiences (Brownell et al. 2013). However, deficits in self-concept development are well-documented in ASD, including atypical use of first person pronouns (Jordan 1996; Lee et al. 1994; Lind and Bowler 2009), reduced understanding of emotions (Ben Shalom et al. 2006; Hill et al. 2004; Silani et al. 2008; Williams and Happé 2010), and impoverished memory for personal facts and events (Bruck et al. 2007; Goddard et al. 2007). Consequently, these impairments in self-understanding may inhibit children’s ability to behave reciprocally in a dynamic sharing interaction. Future research is required to tease apart these theoretical explanations.

Importantly, reduced reciprocity and decreased inequality aversion when sharing could severely impact children’s ability to navigate the social world. The formation and maintenance of positive social relationships requires interpersonal reciprocity (Adamson et al. 2010; Hoffman et al. 2008), and failure to return prosocial behaviour could elicit negative affect in peers and lead to marginalization (Fehr and Gachter 2002; de Quervain et al. 2004). Furthermore, difficulties communicating and understanding others’ mental states may reduce the ability of children with ASD to identify or appraise social feedback indicating how their behaviour is being perceived (Schroeder et al. 2014). These deficits may inhibit the ability of children with ASD to make friends (Bauminger et al. 2008), which in turn exacerbates their vulnerability to bullying (van Roekel et al. 2010). Worryingly, our results suggest that children with ASD might be particularly susceptible to bullies exploiting their lower concern for personal gain and their increased tolerance of unfair behaviour. Moreover, their social naivety and impaired understanding of others’ intentions may inhibit children with ASD from even recognizing when they are being bullied or unfairly manipulated (Sofronoff et al. 2011; van Roekel et al. 2010). These issues may be particularly prominent for children with delayed language development, such as those tested in our study (Zablotsky et al. 2014). We advocate that anti-bullying interventions address these risks by explicitly teaching children the importance of reciprocating prosocial actions, highlighting cues that indicate they are being treated unfairly, teaching prevention strategies, and role-playing good sharing behaviours (Humprhey and Hebron 2015; Sofronoff et al. 2011).

Of course, we must address the limitations of this study. Firstly, it is possible that the observed between-population differences were related to general limitations in cognitive functioning in the ASD sample, or differences in sharing experience associated with chronological age (the ASD group were significantly older than the TD controls). We acknowledge that including a sample of children with delayed intellectual development matched to children with ASD on non-verbal intelligence and chronological age would have eliminated this issue. However, this limitation may be mitigated by (a) the fact that our TD participants responded similarly to TD adults in previous studies (e.g. they offered approximately 40% of the stake in the Ultimatum Game, and made significantly lower offers in the Dictator Game; Camerer 2003), indicating maturity in how they approached the two tasks, (b) TD children’s offers in the Ultimatum and Dictator Games are not influenced by variability in non-verbal intelligence (Han et al. 2012), and (c) offers made by young adults with Down Syndrome, another population with general intellectual difficulties, do not statistically differ from those of TD controls in the Ultimatum Game (Rêgo et al. 2017). Secondly, the Ultimatum and Dictator Games directly encouraged children to share their endowed property with the puppet. It is possible that children with ASD may behave differently in naturalistic social situations that lack the structure and scaffolding of our experimental tasks, or when required to share different kinds of resources (e.g. attachment objects, food, etc). Thus, it would be very interesting to systematically investigate spontaneous sharing in children with ASD and the conditions that are necessary to promote this behaviour in naturalistic contexts (see Brownell et al. 2013). It would also be valuable to explore how differences in sharing behaviour in ASD directly relate to friendship building and bullying. Thirdly, we acknowledge that children’s behaviour within and across games may have been influenced by their relatively unique history with the puppet. The counterbalanced nature of turn orders within games coupled with the puppet’s randomised responses (irrespective of offer fairness) meant that the nature of the interaction varied across participants. Indeed, children’s behaviour in the second game may have been influenced from the outset by the puppet’s actions in game one. Although we have examined the relationship between the child’s and puppet’s behaviour in our reciprocation analyses, much larger sample sizes would be required to identify how each variation of the interaction reliably impacts children’s behaviour.

In summary, our study has shown that children with ASD and TD children offered similar numbers of stickers to a puppet in age-appropriate versions of the classic Dictator and Ultimatum Games. Both groups showed willingness to share equally and neither prioritised self-interest. However, children with ASD were significantly less likely to reciprocate the puppet’s offers (especially in the Ultimatum Game). In naturalistic contexts, failure to reciprocate fair sharing may significantly impact on social cohesion and children’s ability to build relationships (particularly in contexts that depend on the goodwill of a partner). Children with ASD were also much more likely to accept unfair offers, indicating reduced aversion to inequality. We propose that these important differences in sharing behaviour may be linked to broader deficits in social-cognitive development and potentially self-other understanding. These findings inform wider understanding of social interaction deficits that characterise ASD and further specify the nature of their difficulties related to sharing in dynamic social interactions.
